# Aryl hydrocarbon receptor ligands enhance lung immunity through intestinal IKKβ pathways

**DOI:** 10.1186/s12967-019-2043-8

**Published:** 2019-09-05

**Authors:** Tzyy-Bin Tsay, Pei-Hsuan Chen, Lee-Wei Chen

**Affiliations:** 1 Department of Surgery, Kaohsiung Armed Forces General Hospital Zuoying Branch, Kaohsiung, Taiwan; 2 Department of Surgery, Kaohsiung Veterans General Hospital, No.386, Ta-chung 1st Road, Kaohsiung, 813 Taiwan; 3 Department of Biological Sciences, National Sun Yat-Sen University, No.70, Lien-Hai Road, Kaohsiung, 804 Taiwan; 4 Institute of Emergency and Critical Care Medicine, National Yang-Ming University, No.155, Sec.2, Linong Street, Taipei, 112 Taiwan

**Keywords:** Tryptophan, Reactive oxygen species, Alveolar macrophage, Peroxynitrite, NF-κB

## Abstract

**Background:**

Infection by antibiotic-resistant microorganisms is common in intensive care units and has become a global problem. Here, we determined the effect of aryl hydrocarbon receptor (AhR) stimulation on antibiotics-induced systemic defense impairment and its mechanisms.

**Methods:**

C57BL/6 wild-type (WT) mice received combined antibiotics with or without Ahr ligands (tryptophan and indole), or dead *Lactobacillus plantarum* supplementation. The defense mechanisms against *Pseudomonas aeruginosa* infection in the lung were examined.

**Results:**

Antibiotic treatments decreased the phagocytic activity, physiological activity, and the peroxynitrite production of alveolar macrophage (AMs). It also enhanced *P. aeruginosa* pneumonia-induced bacterial counts in the lung. Tryptophan and dead *L. plantarum* supplementation reversed antibiotic-induced intracellular adhesion molecule (ICAM) as well as IL-6 expression, and increased *P. aeruginosa* pneumonia-induced bacterial counts in the lung and increased phagocytic activity and peroxynitrite production of AMs. Moreover, these treatments reversed the antibiotics-induced reduction of Ahr expression, antibacterial proteins, reactive oxygen species (ROS) production, and NF-κB DNA binding activity of the intestinal mucosa and plasma IL-6 levels. *P. aeruginosa* counts increased and phagocytic activity of AMs and myeloperoxidase (MPO) activity decreased in intestinal IKKβ depleted mice. Antibiotics, antibiotic with tryptophan feeding, or antibiotic with dead *L. plantarum* feeding treatments did not change the phagocytic activity and peroxynitrite production of AMs, plasma IL-6 levels, and the expression of Ahr of intestine in intestinal IKKβ depleted mice.

**Conclusion:**

Antibiotic treatment impairs lung immune defenses by decreasing Ahr expression in the intestine and peroyxnitrite production of the AMs. Ahr ligands reverses antibiotic-induced lung defense against bacterial infection through intestinal ROS production and NF-κB activation. The gut is critical in maintaining lung defense mechanism through the intestinal IKKβ pathways.

## Background

Antibiotic use is a crucial part of surgery and treatments such as chemotherapy. However, the antibiotic-resistant infections that are difficult to treat are becoming more and more common. The emergence of these strains is currently a global health crisis. Over 2 million people are infected with antibiotic-resistant bacteria annually in the USA alone, resulting in 23,000 deaths annually, on average [1]. The wide spread use of antibiotics in the intensive care unit (ICU) is an important factor in the emergence of nosocomial infections caused by antibiotic-resistant Gram-negative bacteria [2, 3]. Impairment of the host defenses is a key element in the pathogenesis of infection by these bacteria. However, the effects and the mechanisms of antibiotic-mediated changes in the host defense against bacteria invasion in critical patients are currently still unclear.

The human gastro-intestinal tract is colonized by trillions of microorganisms which encompass hundreds of different species of bacteria and viruses [4, 5]. The intestinal epithelial cells express pattern recognition receptors that protect against antagonistic microbial invasion and maintain epithelial barriers in the presence of commensal microflora [6]. A major downstream effect of toll-like receptor signaling is the activation of nuclear factor-κB (NF-κB) [7]. Paneth cells also limit bacterial penetration to the small intestinal antimicrobial barrier through synthesizing different antimicrobial peptides and proteins, such as enteric α-defensins (known as cryptdins in mice), lysozyme, RegIIIβ, RegIIIγ, RELMβ (resistin-like molecules β), and CRP-ductin [8]. The aryl hydrocarbon receptor (Ahr) is a ligand-dependent transcription factor that is best known for mediating the carcinogenicity xenobiotic ligands. Recent study suggests that Ahr also plays an important physiological role in the immune system [9]. The expression of Ahr is critical for the maintenance, survival, and functioning of Type 3 innate lymphoid cells (ILC 3) [10]. Ahr cooperates with Rorγt to induce the transcription of interleukin-22 (IL-22), which is essential for the clearance of *Citrobacter rodentium* infection [10]. Indole-3-aldehyde, a tryptophan catabolite produced by the microbiota, stimulates the ILCs through the aryl hydrocarbon receptor to induce the ILCs to produce IL-22 [10]. The regulatory mechanisms of the gut-lung axis and the role of the Ahr receptors of the intestinal mucosa in innate lung immunity have remained elusive.

Interleukin-6 (IL-6) is a pleiotropic cytokine involved in both pro-inflammatory and anti-inflammatory responses via the regulation of leukocyte function and apoptosis. Overproduction of IL-6 results in immunological and hematological disorders. In addition, IL-6 induces inflammation by promoting T cell activation, adhesion molecule expression, and influencing leukocyte recruitment [11]. The majority of patients in the ICU are treated with strong antibiotics, which can have pervasive and long-term effects on the intestinal microbiota [12]. In an earlier study, we have demonstrated that antibiotic treatments reduced the total bacteria counts in the terminal ileum, but increased the translocation of injected pathogenic *Klebsiella pneumoniae* due to a reduction in the mucosal bacterial killing activity and the expression of nondefensin proteins [13]. Recently, we also showed that dead *L. salivarius* or FOS feeding reversed antibiotic-induced lung defense impairment through the intestinal ROS/MyD88 pathways [14]. The gut microbiota has been proven to enhance primary alveolar macrophage (AM) function and plays a protective role in the host defense against pneumococcal pneumonia [15]. We hypothesize that Ahr ligands or dead *Lactobacillus plantarum* could be used to strengthen the lung defense against *Pseudomonas aeruginosa* (PA)-induced pneumonia in ICU patients through ROS production and NF-κB activation from the intestine. Here, mice were used as a model in antibiotic treatment with or without PA-induced pneumonia to study the effects and mechanisms of the antibiotic treatment on lung defense mechanisms. There are three main objectives in this study: (1) To determine the effects and mechanisms of antibiotic treatments on lung defense mechanisms. (2) To determine whether Ahr ligands could reverse the inhibitory effects of antibiotics on lung defense. (3) To examine the involvement of Ahr and IKKβ of the intestine in the regulatory mechanisms of the gut on lung defense. In ICU patients, oral supplementation of Ahr ligands to reverse lung defense impairment caused by antibiotic treatments can potentially be a useful therapeutic treatment in the future.

## Methods

### Animals

We obtained specific pathogen-free (SPF) C57BL/6J mice from the National Laboratory Breeding and Research Center (NLBRC, Taipei, Taiwan). Specific pathogen-free intestinal epithelial cell-specific, IKKβ-deficient (*Vil*-*Cre/Ikkβ*^*F/Δ*^) mice and control (*Ikkβ*^*F/Δ*^) mice were generated from the same back ground and transferred from Dr. Karin’s lab. (University of California, San Diego). Before the experiment, the animals were maintained in a temperature-controlled room and fed a standard diet of Purina mouse chow with water allowed ad libitum for at least 1 week before the experiment. All animal procedures were in compliance with the regulations on animals used for experimental and other scientific purposes approved by the Kaohsiung Veterans General Hospital Animal Experiments Committee.

### Experimental design

#### Experiment 1

To simulate combined antibiotic treatment use in clinical patients, we used a broad-spectrum antibiotic treatment regimen where the mice (C57BL/6) received combined intramuscular injections (100 μl) daily. The injections are composed of a blend of different antibiotics: ampicillin (1000 mg/L; Bio Basic Inc.), vancomycin (500 mg/l; Hospira), and metronidazole (1000 mg/l; Sigma) [13]. The mice were randomly divided into the following four groups: (1) Control, (2) Tryptophan, (3) Indole, and (4) Lactobacillus. Tryptophan (Sigma-Aldrich) or indole (Alfa Aesar) were administered to mice in drinking water to stimulate aryl hydrocarbon receptors (Ahr) in the intestine for 2 days before sacrifice in order to examine the effects of Ahr ligands on antibiotic treatment-induced lung defense impairment. To determine if increasing specific groups of intestinal commensal microbiota (i.e., *Lactobacillus* or *Bifidobacteria*) can lead to the improvement and prevention by tryptophan supplementation, mice were fed dead *L. plantaris* (2 × 10^8^ CFU/ml) for 6 days. Other C57BL/6 mice were randomly divided into the following five groups: Group I received intramuscular injections of normal saline daily over 6 days (control group); Group II received intramuscular injections of antibiotics daily for 6 days; Group III received antibiotics injections with intramuscular injections of antibiotics daily over 6 days with tryptophan feeding daily for 2 days; Group IV received antibiotics injections over 6 days and were fed dead *Lactobacillus plantaris* (2 × 10^8^ CFU/ml) daily over 6 days; Group V received antibiotics injections with indole feeding daily over 2 days and antibiotics injection daily over 6 days. At the time of sacrifice, the ileum was harvested for Western Blotting, reactive oxygen species assessment, and bacterial killing activity. Phagocytic activity and tumor necrosis factor (TNF-α) production of AMs were measured in another set of animals.

#### Experiment 2

To investigate the effects of antibiotic treatment on PA-induced neutrophil infiltration and bacterial counts in the lung and mechanisms involved, combined antibiotics were administered to mice over 6 days. C57BL/6 mice were randomly divided into four groups as experiment 1. The mice received PA intra-tracheal instillations, and were sacrificed 8 h following the instillation. The lung tissue was harvested for PA culturing.

#### Experiment 3

*Vil*-*Cre/Ikkβ*^*F/Δ*^ mice and *Ikkβ*^*F/Δ*^ mice were used to investigate the involvement of IKK activity of the intestinal mucosa in PA-induced neutrophil infiltration in the lung. The mice were randomly divided into two groups: Group I received intramuscular injections of normal saline daily over 6 days (control group); Group II received intramuscular injections consisting of combined antibiotics daily over 6 days. The animals received PA intra-tracheal instillations and were sacrificed 8 h following the instillation. The lung tissue was harvested for PA culturing and myeloperioxidase activity assay.

#### Experiment 4

*Vil*-*Cre/Ikkβ*^*F/Δ*^ mice and *Ikkβ*^*F/Δ*^ mice were used to assess the involvement of IKKβ activity in the intestine and PA-induced neutrophil infiltration in the lung. The *Vil*-*Cre/Ikkβ*^*F/Δ*^ mice were randomly divided into four groups as experiment 1. There were six replicates in each treatment group (n = 6). Peroxynitrite production, phagocytic activity of AMs, and plasma IL-6 levels were examined.

### Tryptophan, dead *Lactobacillus plantaris*, and indole feeding

To investigate the effects of Ahr ligands on antibiotic treatment-induced intestinal dysbiosis and systemic defense impairment, tryptophan (i.e., trp; 2 mg in 200 μl water was administered orally by gavage the third and fourth days), indole (475 μg in 250 μl was administered orally by gavage on the third and fourth days), or dead *L. plantaris* (CECT 5713, 2 × 10^8^ CFU/ml in drinking water over 6 days) [16] were administered the mice. The animals were given access to water via a bottle ad libitum. A fresh batch of water containing tryptophan, dead *L. plantaris*, or indole was provided daily. The dead bacteria were completely suspended in the water where there was no precipitation of bacteria in the bottle. The control group was provided drinking water without the supplementation of trp, dead *L. plantaris*, or indole.

### Induction of *P. aeruginosa* pneumonia

Mice were anesthetized with ketamine hydrochloride (100 mg/kg intramuscularly, Veterinary Laboratories, Wyeth-Ayerst Canada Inc., Mississauga, ON, Canada) and xylazine (5 mg/kg intramuscularly, Bayer Inc., Mississauga, ON, Canada). The trachea was surgically exposed and 50 µl (1.0 × 10^7^ CFU *P. aeruginosa*) were instilled via an angiocatheter through the trachea.

### Bacterial counts of lung after exposure to bacteria

Animals were sacrificed by intra-peritoneal injection of ketamine (80 mg/kg) and xylazine (10 mg/kg) 18 h following PA intra-tracheal injection. The bacterial clearance between the different treatments was compared. The whole lung was excised under sterile conditions and washed with 10 ml of sterile cold saline. The viable bacteria counts of homogenized lung and blood material were determined after an 18-h culturing at 37 °C on TSB–agar plates. Data were expressed as CFU/ml.

### Polymerase chain reaction (PCR) and quantitative real-time PCR

Total RNAs were isolated from lung using total RNA Miniprep Purification Kits (GeneMark). Reverse transcription-generated cDNAs were amplified using PCR. Sets of ICAM and IL-6 primers were obtained from references [17, 18], and one pair of primers for GAPDH gene as a control.

For the real-time PCR reactions, 200 ng of the cDNA template was added to 20 μl of mixture containing 12.5 μl of 2× KAPA SYBR^®^ FAST qPCR Master Mix (Kapa Biosystems), 2.5 μl of each sense and anti-sense primers (25 μM) and 5 μl of sterile water. The amplification was performed in a StepOnePlus™ Real-Time PCR System (Applied Biosystems 7300).

### Phagocytic activity of alveolar macrophages (AMs)

In the lung, alveolar macrophages (AMs) represent the first line of defense against pathogens such as *P. aeruginosa*. In the presence of pathogens, the lung epithelial cells promote neutrophil sequestration which may cause injury to the lung as well as activate the immunological response [19]. The AMs were collected and re-suspended in Hank’s Balanced Salt Solution (HBSS) (10^6^ cells/ml). After 5 min of pre-incubation, the cell suspension was incubated with *P. aeruginosa* (10^8^ cells/ml) at 37 °C for 1 h with shaking. The culture was then centrifuged at 200×*g* for 10 min. The pellet with the cells was removed and the *P. aeruginosa* in the supernatant was counted [20, 21].

### Assessment of activity of AMs by ex vivo stimulation

Bronchoalveolar lavage (BAL) was conducted to harvest AMs from adult mice with Tris saline solution containing 0.25 mM EDTA and EGTA. Cells were re-suspended in RPMI 1640 medium in a final concentration of 1 × 10^5^ cells/ml. Cells were then cultured in 96-well microtiter plates for 2 h and washed with RPMI 1640 to remove the nonadherent cells [22]. The adherent monolayer of cells was stimulated with 5 µg/ml of lipopolysaccharide (LPS from *Escherichia coli* O26:B6 Sigma-Aldrich) or RPMI 1640 for 4 h. The supernatants were collected and stored at − 70 °C until the TNF-α assay.

### Peroxynitrite production of AMs (123-DHR oxidation assay of AMs)

Peroxynitrite is a potent macrophage-derived oxidizing cytotoxin that damages and kills invading pathogens. The collected AMs were adjusted to 3.0 × 10^6^ ml^−1^ in RPMI + 10% fetal bovine serum, and 100 μl of HBSS (without phenol red) containing 25 μM of 1,2,3-dihydrorhodamine (Invitrogen, Eugene, OR), a peroxynitrite-detecting dye was added. The cells were stimulated with 1 μg/ml of *E. coli* LPS (Sigma). Peroxynitrite levels were measured using a fluorescence plate reader (Synergy HT Biotek, Winooski, VT) every 15 min for 75 min using excitation and emission wavelengths of 485 nm and 530 nm, respectively. The fluorescence due to auto-oxidation of 123-DHR was subtracted from the original measurements [23].

### Western immunoblots

The Ahr, RELMβ, and CRP-ductin were identified by mouse monoclonal antibodies (*R&D Systems*); the CRP-ductin were identified by mouse monoclonal, rabbit polyclonal and goat polyclonal antibodies, respectively (Santa Cruz Biotechnology Inc.).

### Electrophoretic mobility shift assay for NF-κB

The intestinal mucosa was harvested by centrifugation and was used to prepare the nuclear extract as described previously [24]. The consensus and control oligonucleotides (Santa Cruz Biotechnology Inc.) were labeled by polynucleotide kinase, the NF-κB consensus sequence was 5′AGTTGAGGGGAC-TTTCCCAGGC3′ (1.75 pmol/l). The samples were analyzed on a 4% polyacrylamide gel and the gel was dried and visualized by using autoradiography.

### ROS levels in the intestinal mucosa

The levels of ROS in the intestinal mucosa were analyzed by 10 mM DCFDA fluorescent dye (Sigma), which was added into the suspension of intestinal mucosa for cultivation. DCFDA is deacetylated by cellular esterases to a non-fluorescent compound, which is later oxidized by ROS into 2ʹ,7ʹ-dichlorofluorescein (DCF). DCF is detected by fluorescence spectroscopy with excitation and emission spectra of 495 nm and 529 nm, respectively [25].

### Enzyme-linked immunosorbent assay (ELISA)

The mouse ELISA kit (eBioscience) was used for IL-6 assay. The blood was centrifuged at 1000×*g*, 4 °C for 15 min and the serum was collected for use. The ELISA plates were coated with 100 μl capture antibody per well at 4 °C overnight. After appropriate wash, 200 μl of assay dilution buffer was added per well for blocking at room temperature for 1 h. The sample and serial dilutions of standards were added to the plate and incubated at 4 °C overnight. After coating with detection antibody, avidin-HRP was added and incubated at room temperature for 30 min. The substrate 3,3′,5,5′-tetramethylbenzidine (TMB) was added and incubated for 15 min. Finally, 2 N H_2_SO_4_ was added to stop the reaction and absorbance at 450 nm was measured using an ELISA reader.

### Neutrophil infiltration in the lungs

Lung myeloperoxidase (MPO) activity has been used as a marker of lung neutrophil infiltration [26]. Lung tissues were weighed and homogenized in 50 mM potassium phosphate buffer (pH 6.0) with 0.5% hexadecyltrimethyl-ammonium bromide. Homogenates were centrifuged at 9500×*g*, 4 °C for 10 min. An aliquot (60 μl) of supernatants was added to 939 μl of potassium phosphate buffer with 16.7 mg/ml of O-dianisidine and 0.5% hydrogen peroxide. The rate of change in absorbance at 460 nm was measured over 2 min. One unit of MPO activity is defined as the amount of enzyme that reduces 1 μmol of peroxide per min and the data were expressed as units per gram of lung tissue (Units/g tissue).

### Statistical analysis

All data are analyzed by one-way analysis of variance or T-test analysis of variance (ANOVA), followed by Turkey’s Multiple Comparison Test. All values in the figures and text are expressed as mean ± standard error of the mean. P values of less than 0.05 are considered to be statistically significant.

## Results

### Oral supplementation with Trp, or dead *L. plantaris* decreases *P. aeruginosa* pneumonia-induced bacterial counts in the lung under antibiotic treatment

**Fig. 1:**
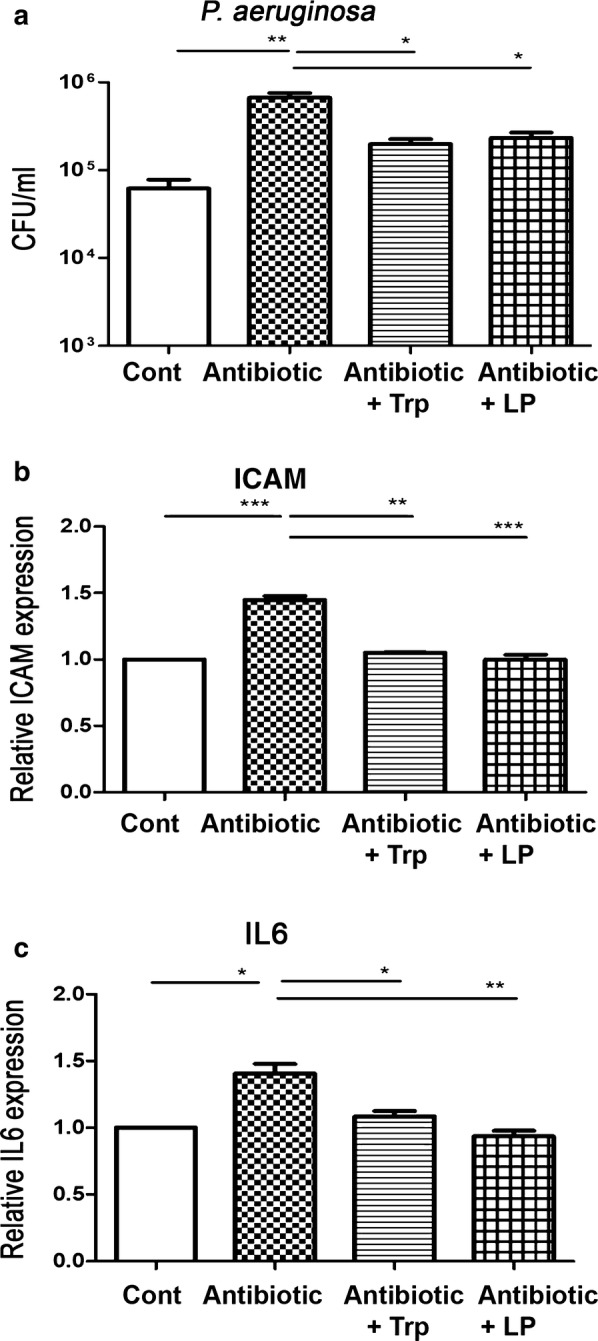
Oral supplementation with Trp, or dead *L. plantaris* decreases *P. aeruginosa* pneumonia-induced bacterial counts and ICAM and IL-6 expression of the lung under antibiotic treatment. C57BL/6 male mice received a combined intramuscular injection of antibiotics: ampicillin, vancomycin, and metronidazole for 6 days. Tryptophan, indole, or dead *L. plantaris* were given to mouse. **a** Intramuscular combined antibiotics with or without Trp or dL.P. supplementation were given to mice for 6 days. The trachea of mice was surgically exposed and 50 μl (1.0 × 10^7^ CFU *P. aeruginosa*) were instilled via an angiocatheter through the trachea to induce *P. aeruginosa* pneumonia. Data are presented as mean ± SEM. **b** Antibiotic treatment significantly increased ICAM and mRNA expression in the lung and Trp, or dead *L. plantaris* feeding decreased it. Data are presented as mean ± SEM. **c** Antibiotic treatment increased IL-6 mRNA expression in the lung and Trp, or dead *L. plantaris* feeding decreased it. Data are presented as mean ± SEM; PA, *P. aeruginosa*; *P < 0.05, **P < 0.01

To define the effects of antibiotic treatment on lung immune defenses against *P. aeruginosa* infection, we examined the bacterial counts from the lung in WT mice receiving *P. aeruginosa* intra-tracheal injection. Antibiotic pretreatment significantly increased PA-induced bacterial counts compared to those in the saline control treatment (665,000 ± 92,000 vs. 62,000 ± 16,000 CFU/ml) (Fig. 1a). Trp and dead *L. plantaris* feeding following antibiotic treatment significantly reduced PA bacterial counts compared to the antibiotic only group (198,000 ± 26,000, 232,000 ± 37,000, vs. 665,000 ± 92,000 CFU/ml). These results indicate that the antibiotic treatments reduced lung defense against infection by *P. aeruginosa*. On the other hand, tryptophan and dead *L. plantarum* feeding was able to restore the defense function.

### Oral supplementation with Trp, or dead *L. plantaris* decreases *P. aeruginosa* pneumonia-induced ICAM and IL-6 expression in the lung under antibiotic treatment

In order to evaluate the effect of antibiotic treatment on PA-induced lung inflammation, we assessed the ICAM and IL-6 mRNA expression in the lung tissue in WT mice. Antibiotic pretreatment significantly increased ICAM and IL-6 mRNA expression of compared to the control treatment (Fig. 1b, c). Trp and dead *L. plantaris* feeding after antibiotic treatment significantly decreased ICAM and IL-6 expression compared to in the antibiotic only group. These results indicate that antibiotic treatments increased lung inflammation. Conversely, tryptophan and dead *L. plantarum* feeding reversed this effect.

### Oral supplementation with Trp, indole, and dead *L. plantaris* improves phagocytic activity of alveolar macrophages under antibiotic treatment

**Fig. 2:**
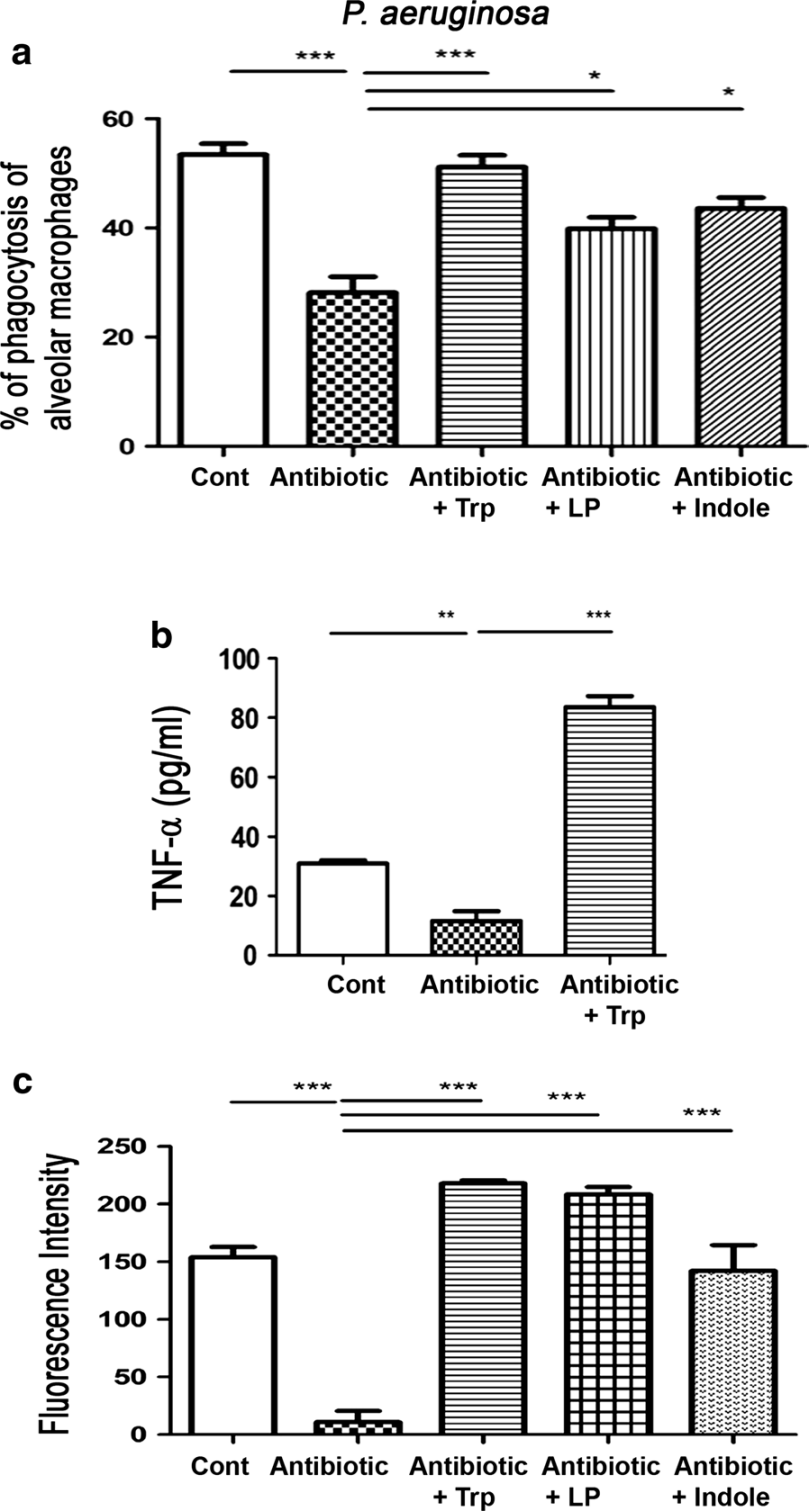
Oral supplementation with Trp, or dL.P. increases phagocytic activity and peroxynitrite production of alveolar macrophages under antibiotic treatment. C57BL/6 male mice received a combined intramuscular injection of antibiotics: ampicillin (1000 mg/l), vancomycin (500 mg/l), and metronidazole (1000 mg/l) at 100 μl/day for 6 days. Trp (250 mg per day) or dead *L. plantarum* (2 × 10^8^ CFU/ml) was given in drinking water for 6 days. **a** Antibiotic treatment for six days decreased *P. aeruginosa* killing activity of alveolar macrophage and oral feeding of trp, indole, or dead *L. plantarum* increased it. AMs were collected and resuspended in HBSS as 10^6^ cells/ml. After 5 min of pre-incubation, the cell suspension was incubated with *P. aeruginosa* (10^8^/ml). The cells were removed and *P. aeruginosa* in the supernatant was counted. Data are presented as mean ± SEM. **b** Antibiotic treatment for 6 days decreased AMs activity. Tryptophan feeding in mice receiving antibiotic treatment significantly increased the AMs activity. AMs were harvested from adult mice by bronchoalveolar lavage (BAL). Cells were then cultured in 96-well microtiter plates to remove nonadherent cells. Adherent monolayer cells were stimulated with 0.5 or 5 μg/ml of LPS (from *Escherichia coli* O26:B6 Sigma-Aldrich). Supernatants were assayed for TNF-α. Data are presented as mean ± SEM. **c** Intramuscular combined antibiotic was given to mice for 6 days and AMs were purified from BALF for peroxynitrite production assay. The collected AMs were cultured with phenol red containing 25 μM of 1,2,3-dihydrorhodamine. The cells were stimulated with *E. coli* LPS. Peroxynitrite was measured every 15 min for 75 min using excitation and emission wavelengths of 485 and 530 nm, respectively. Data are presented as mean ± SEM; PA, *P. aeruginosa*; *P < 0.05, **P < 0.01, ***P < 0.001

To further evaluate the effects of antibiotic treatments on the lung defense mechanism, and whether Ahr ligands could reverse the antibiotic treatment-induced reduction of phagocytic activity of AMs, the mice were fed tryptophan, indole, or dead *L. plantarum* after the antibiotic treatment. Antibiotic treatment with or without Trp or dead *L. plantarum* feeding did not change the number of alveolar macrophages as compared with control group. Antibiotic treatment over 6 days significantly decreased the activity of the AMs in killing *P. aeruginosa* down to nearly 50% in comparison with the control (24.83 ± 3.1 vs. 53.41 ± 1.99%, respectively). Oral feeding of Trp, dead *L. plantarum*, or indole receiving antibiotic treatment significantly increased the phagocytic activity of the AMs twofold compared to those in mice receiving only antibiotics (51.13 ± 2.20, 39.20 ± 3.40, 43.60 ± 2.00 vs. 24.83 ± 3.10%, respectively) (Fig. 2a). These results indicate that the antibiotic treatment reduced the phagocytic activity of AMs on *P. aeruginosa.* On the other hand, Ahr ligands and dead *L. plantarum* feeding was able to restore the activity.

### Oral supplementation with Trp increases activity of AMs under antibiotic treatment

Antibiotic treatment significantly decreased AMs activity down to around 30% compared to the control (30.99 ± 1.07 vs. 11.58 ± 3.29%, respectively). For mice receiving antibiotic treatment, tryptophan feeding significantly increased the AMs activity sixfold compared to the antibiotic only group (83.53 ± 3.76 vs. 11.58 ± 3.29%, respectively) (Fig. 2b). These results suggest that the antibiotic treatment reduces AM activity; however tryptophan feeding is able to reverse it. These results suggest that the antibiotic treatment reduces AM activity; however tryptophan feeding not only restores but also increases this activity.

### Oral supplementation of trp, indole, and dead *L. plantaris* increases peroxynitrite production of AMs under antibiotic treatment

Superoxide and nitric oxide are essential components for the synthesis of microbicidal compounds (e.g., peroxynitrite) in macrophages [23]. To further examine the mechanisms of antibiotic treatment-induced impairment in lung defenses, we harvested AMs from the lungs and examined their production of peroxynitrite. Antibiotic treatment significantly reduced the peroxynitrite production of AMs in WT mice by 93% compared to the control group (153.89 ± 8.84 vs 10.83 ± 9.69, respectively) (Fig. 2c). Moreover, for mice receiving antibiotic treatment, tryptophan, indole, and dead *L. plantarum* feeding significantly increased the peroxynitrite production of AMs 20-, 19-, and 13-fold as compared to the antibiotic treatment group, respectively (i.e., 218.09 ± 2.74, 208.39 ± 6.59, 142.27 ± 22.23 vs. 10.83 ± 9.69, respectively) (Fig. 2c). These results indicate that antibiotic treatments decrease the peroxynitrite production of AMs. Conversely, tryptophan, indole, and dead *L. plantarum* restores the peroxynitrite production.

### Antibiotic treatment reduces the expression of the aryl hydrocarbon receptor, RELMβ, and CRP-ductin of the intestine while tryptophan and dead *L. plantaris* feeding restores them

**Fig. 3:**
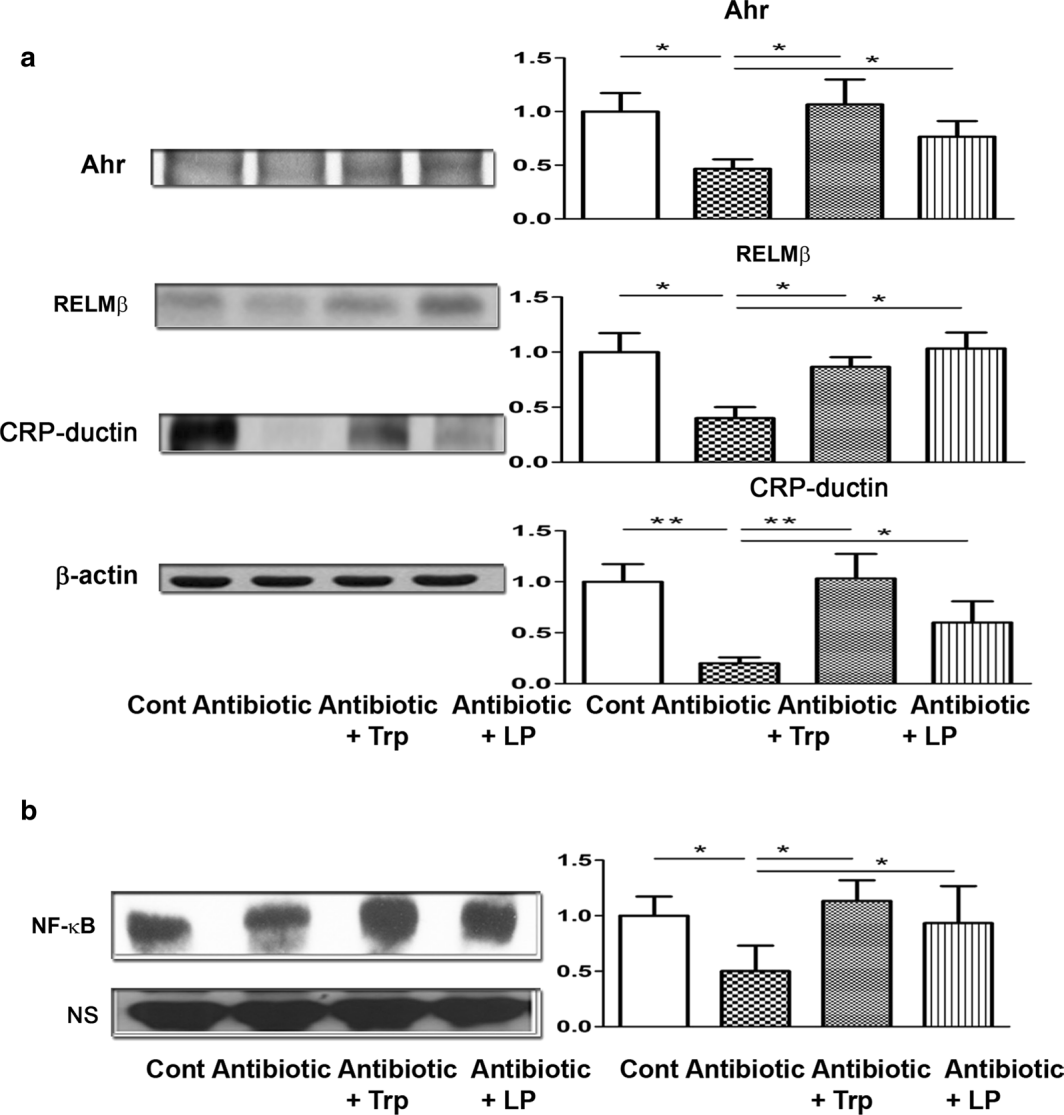
Oral supplementation with Trp, or dL.P. increases aryl hydrocarbon receptor and antibacterial protein and NF-κB DNA binding activity of the intestinal mucosa under antibiotic treatment. **a** Antibiotic treatment significantly decreased aryl hydrocarbon receptor and antibacterial protein expression of the intestinal mucosa including RELMβ and CRP-ductin as compared with the control mice. Oral feeding of tryptophan or dead *L. plantarum* feeding increased Ahr, RELMβ, and CRP-ductin expression in the intestinal mucosa in WT mice as compared with the antibiotic treatment group. The Ahr, RELMβ, and CRP-ductin were identified by western immunoblots. Data are presented as mean ± SEM. **b** Antibiotic treatment significantly decreased the NF-κB DNA binding activity of the intestinal mucosa as compared to that of the control group. Oral feeding of tryptophan or dead *L. plantarum* feeding significantly increased the NF-κB DNA binding activity of the intestinal mucosa as compared with the antibiotic group. Data are presented as mean ± SEM; *P < 0.05, **P < 0.01

To examine the effects of antibiotic treatment on the expression of aryl hydrocarbon receptor and the antibacterial protein of the intestinal mucosa, the protein expression of Ahr, RELMβ, and CRP-ductin of the intestinal mucosa was examined in mice from the different treatments (Fig. 3a). Antibiotic treatment significantly decreased aryl hydrocarbon receptor and antibacterial protein expression of the intestinal mucosa, including RELMβ and CRP-ductin, compared with the control. Oral feeding of tryptophan and dead *L. plantarum* feeding significantly increased Ahr, RELMβ, and CRP-ductin expression of the intestinal mucosa in WT mice compared to the antibiotic treatment only group (Fig. 3a). These results indicate that antibiotic treatment reduces the expression of antibacterial proteins as well as Ahr of the intestinal mucosa. Conversely, tryptophan and dead *L. plantarum* feeding restores the protein expression.

### Antibiotic treatment reduces NF-κB DNA binding activity in the intestinal mucosa while tryptophan or dead *L. plantaris* feeding reverses the reduction

The NF-κB DNA binding activity of the intestinal mucosa was examined to evaluate the effects of antibiotic treatment. The antibiotic treatment significantly reduced the NF-κB DNA binding activity compared to the control (Fig. 3b). Oral feeding of tryptophan and dead *L. plantarum* feeding significantly increased the NF-κB DNA binding activity of the intestinal mucosa compared to the antibiotic only control group (Fig. 3b). These results indicate that the antibiotic treatment reduces NF-κB DNA binding activity of the intestinal mucosa, while tryptophan and dead *L. plantarum* feeding restores the function.

### Antibiotic treatment decreases the ROS levels of the intestinal mucosa while tryptophan, indole, and dead *L. plantarum* feeding reverses the effect

**Fig. 4:**
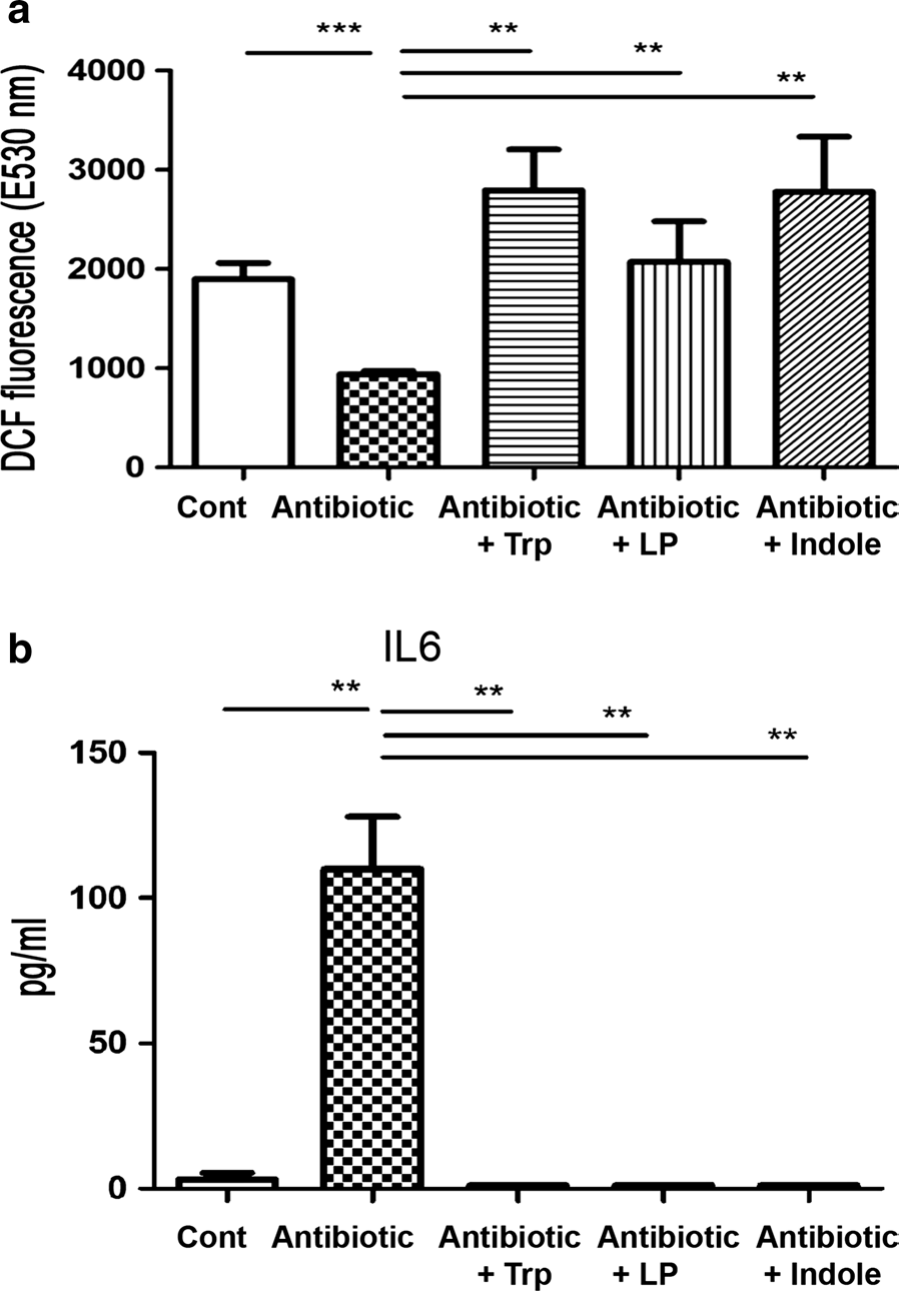
Oral supplementation with Tryptophan, dead L.P., or indole increases ROS levels of the intestinal mucosa and decreases IL-6 levels in the plasma under antibiotic treatment. **a** Intramuscular combined antibiotic was given to mice for 6 days and ROS production in the intestinal mucosa was examined with the production of DCFDA. The levels of ROS in the intestinal mucosa were analyzed by DCFDA fluorescent dye, which was added into the suspension of intestinal mucosa for the cultivation. DCFDA is oxidized by ROS into 2ʹ7ʹ-dichlorofluorescein (DCF). DCF is detected by fluorescence spectroscopy with excitation and emission spectra of 495 nm and 529 nm, respectively. Data are presented as mean ± SEM. **b** Antibiotic treatment significantly increased the IL-6 levels in the plasma by 200-fold as compared to that in the control group. Tryptophan or dead L.P. feeding in mice receiving antibiotic treatment decreased the plasma IL-6 levels as compared to those in the antibiotic treatment group. The mouse ELISA kit (eBioscience) was used for IL-6 assay. The blood was centrifuged at 1000×*g*, 4 °C for 15 min and the serum was collected for use. Data are presented as mean ± SEM; DCF, 2ʹ7ʹ-dichlorofluorescein; *P < 0.05, **P < 0.01

To examine the involvement of intestinal ROS in antibiotics-induced lung defense impairment, we examined the dichlorofluorescin diacetate (DCFDA) levels of the intestinal mucosa in mice from different treatment groups. Antibiotic treatment significantly decreased the DCFDA levels of the intestinal mucosa by 52% compared to the control (920.2 ± 36.12 vs. 2236 ± 46.85 at E350 nm, respectively) (Fig. 4a). In mice receiving the antibiotic treatment, tryptophan, indole, and dead *L. plantarum* feeding significantly increased the DCFDA levels of the intestinal mucosa 3, 2.65, and 3.38-fold compared to the antibiotic group, respectively (i.e., 2796 ± 407.8, 2438 ± 314, 3117 ± 625.3 vs. 920.2 ± 36.12 E350 nm, respectively) (Fig. 4a). These results indicate that antibiotic treatment decreases ROS levels of the intestinal mucosa. Conversely, tryptophan, indole, and dead *L. plantarum* restores the ROS levels.

### Antibiotic treatment increases plasma IL-6 levels while tryptophan, dead *L. plantarum*, and indole feeding reduces them

To study the involvement of IL-6 in antibiotics-induced lung inflammation, we examined plasma IL-6 levels in the different treatment groups. Antibiotic treatment significantly increased the plasma IL-6 levels 34-fold as compared to that in the control group (110.0 ± 17.96 vs. 3.23 ± 2.23 E350 nm, respectively) (Fig. 4b). Tryptophan, dead *L. plantarum*, and indole feeding in mice receiving antibiotic treatment significantly decreased the IL-6 levels in the plasma compared to those in the antibiotic treatment only group (0, 0, 0 vs. 110.0 ± 17.96 E350 nm, respectively) (Fig. 4b). These results indicate that antibiotic treatment increases IL-6 levels in the plasma while tryptophan, indole, or dead *L. plantarum* feeding reverses this effect.

### IKKβ of intestine is critical in regulating lung defense against *P. aeruginosa* infection

**Fig. 5:**
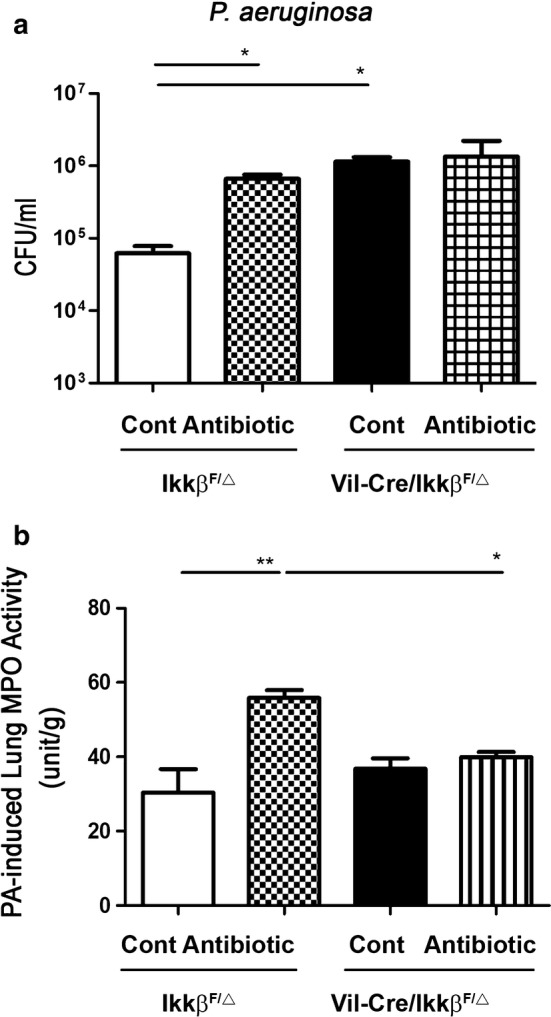
Effect of depletion of IKKβ in intestine on lung against *P. aeruginosa* pneumonia-induced bacterial counts and MPO activity of the lung. **a** Intramuscular combined antibiotic was given to *Vil*-*Cre/Ikkβ*^*F/Δ*^ or *Ikkβ*^*F/Δ*^ mice for 6 days. The trachea of mice was surgically exposed and 50 μl (1.0 × 10^7^ CFU *P. aeruginosa*) were instilled via an angiocatheter through the trachea to induce *P. aeruginosa* pneumonia and neutrophil infiltration in the lungs was examined. *P. aeruginosa* intra-tracheal injection induced a significant increase of bacterial counts in the lung in *Vil*-*Cre/Ikkβ*^*F/Δ*^ mice as compared with that in *Ikkβ*^*F/Δ*^ mice. Antibiotic pretreatment significantly increased twofold of *P. aeruginosa*-induced bacterial counts in the lung in *Ikkβ*^*F/Δ*^ mice. Data are presented as mean ± SEM. **b** Antibiotics treatment significantly increased twofold of *P. aeruginosa*-induced MPO activity of lung in *Ikkβ*^*F/Δ*^ as compared with the control group. Antibiotic pretreatment did not increase *P. aeruginosa*-induced MPO activity in the lung in *Vil*-*Cre/Ikkβ*^*F/Δ*^ mice. Lung tissues were weighed and homogenized in 50 mM potassium phosphate buffer (pH 6.0) with 0.5% hexadecyltrimethyl- ammonium bromide. Homogenates were centrifuged at 9500×*g*, 4 °C for for 10 min. An aliquot (60 μl) of supernatants was added to 939 μl of potassium phosphate buffer with 16.7 mg/ml of O-dianisidine and 0.5% hydrogen peroxide. Data are presented as mean ± SEM. *P < 0.05, **P < 0.01

To evaluate the role of IKKβ of the intestinal mucosa in lung defense against infection by PA, we examined the PA bacterial growth of the lung of *Vil*-*Cre/Ikkβ*^*F/Δ*^ and *Ikkβ*^*F/Δ*^ mice receiving *P. aeruginosa* via intra-tracheal injection. *P. aeruginosa* intra-tracheal injection significantly increased the bacterial counts of the lung in gut specific *Vil*-*Cre/Ikkβ*^*F/Δ*^ mice compared to the *Ikkβ*^*F/Δ*^ mice (1,140,000 ± 170,000 vs. 64,000 ± 18,700 CFU/ml, respectively) (Fig. 5a). Antibiotic pretreatment significantly increased the PA-induced bacterial counts in the lungs of *Ikkβ*^*F/Δ*^ mice (Fig. 5a). However, the antibiotic pretreatment did not increase PA-induced bacterial counts in the lungs of *Vil*-*Cre/Ikkβ*^*F/Δ*^ mice compared to the control group (Fig. 5a). These results indicate that the IKKβ of the intestine is critical in regulating lung immune defense against infection by *P. aeruginosa*. The inhibitory effect of the antibiotic treatment on lung defense occurs through IKKβ of the intestine.

### IKKβ of intestine is critical in antibiotics-induced neutrophil infiltration in lung

To further evaluate the role of IKKβ of the intestine in neutrophil infiltration in the lung, we examined the myeloperoxidase (MPO) activity of the lung in *Vil*-*Cre/Ikkβ*^*F/Δ*^ and *Ikkβ*^*F/Δ*^ mice receiving intra-tracheal injection of *P. aeruginosa* following antibiotics treatment. The antibiotics treatment significantly increased the PA-induced MPO activity in the lung by twofold in *Ikkβ*^*F/Δ*^ compared to the control (54.34 ± 2.24 vs. 30.40 ± 6.31 unit/g, respectively) (Fig. 5b). These results indicate that the antibiotics treatment increased PA-induced neutrophil infiltration in the lung. However, the antibiotic pretreatment did not increase PA-induced MPO activity in the lungs of *Vil*-*Cre/Ikkβ*^*F/Δ*^ mice compared to the control group (39.94 ± 1.34 vs. 36.77 ± 2.83 unit/g, respectively) (Fig. 5b). These results indicate that IKKβ of the intestine plays an important role in neutrophil infiltration in the lung.

### IKKβ of intestine is critical in the maintenance of *P. aeruginosa* killing activity of AMs

**Fig. 6:**
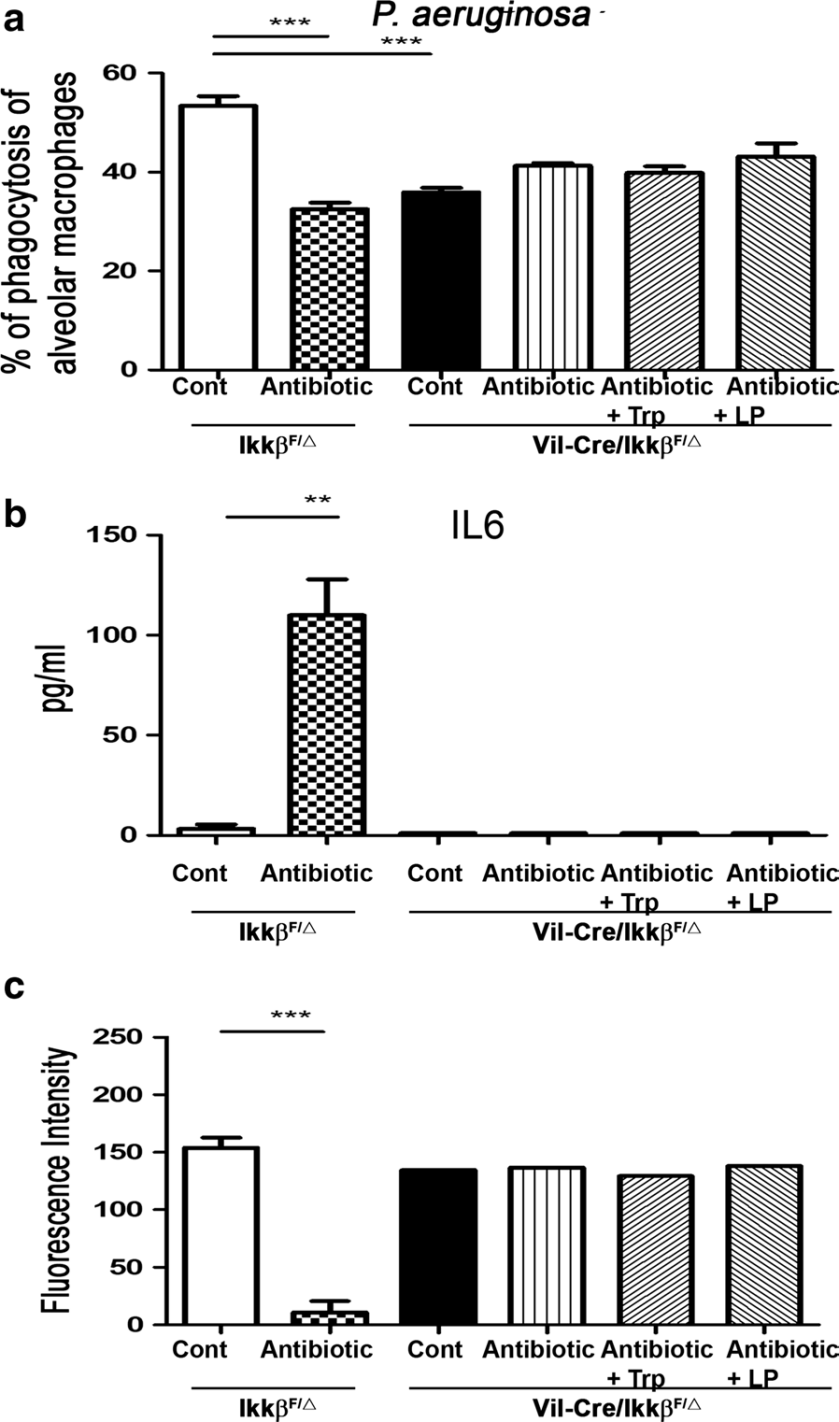
IKKβ in intestine is critical in the effects of antibiotic treatment on the phagocytic activity and peroxynitrite production of AMs, and IL-6 levels in plasma. **a** IKKβ of intestine is critical in the phagocytic activity of AMs and tryptophan, or dead *L. plantarum* feeding-induced phagocytic activity of AMs. Data are presented as mean ± SEM. **b** Antibiotic treatment significantly increases IL-6 plasma levels of *Vil*-*Cre/Ikkβ*^*F/Δ*^ mice. Antibiotics, antibiotic with Tryptophan, or antibiotic with dead *L. plantarum* did not change IL-6 plasma levels in *Vil*-*Cre/Ikkβ*^*F/Δ*^ as compared with the control group in *Ikkβ*^*F/Δ*^ mice. The mouse ELISA kit (eBioscience) was used for IL-6 assay. The blood was centrifuged at 1000×*g*, 4 °C for for 15 min and the serum was collected for use. Data are presented as mean ± SEM. **c** Peroxynitrite production of AMs of *Vil*-*Cre/Ikkβ*^*F/Δ*^ mice did not demonstrate a significant change as compared with that in *Ikkβ*^*F/Δ*^ mice. Antibiotics, antibiotic with Tryptophan, or antibiotic with dead *L. plantarum* did not change peroxynitrite production of AMs in *Vil*-*Cre/Ikkβ*^*F/Δ*^ as compared with that *Ikkβ*^*F/Δ*^ mice. The collected AMs were cultured with phenol red containing 25 μM of 1,2,3-dihydrorhodamine. The cells were stimulated with *E. coli* LPS. Peroxynitrite was measured every 15 min for 75 min using excitation and emission wavelengths of 485 and 530 nm, respectively. Data are presented as mean ± SEM; *P < 0.05, **P < 0.01

The *P. aeruginosa* killing activity of the AMs in *Vil*-*Cre/Ikkβ*^*F/Δ*^ and *Ikkβ*^*F/Δ*^ mice was examined to assess the involvement of IKKβ of the intestine in lung immune defense against *P. aeruginosa* infection. When the suspension was incubated with the AMs of control *Ikkβ*^*F/Δ*^ mice, 55% of the bacteria in the suspension was killed. The antibiotic treatment significantly decreased the phagocytic activity of AMs in *Ikkβ*^*F/Δ*^ compared to those in the control. Only 36% of the bacteria were killed when incubated with AMs of *Vil*-*Cre/Ikkβ*^*F/Δ*^ mice. The antibiotic only, antibiotic with tryptophan feeding, and antibiotic with dead *L. plantarum* feeding treatments did not change the phagocytic activity of AMs in *Vil*-*Cre/Ikkβ*^*F/Δ*^ mice compared to the control group in *Vil*-*Cre/Ikkβ*^*F/Δ*^ mice (Fig. 6a). These results indicate that IKKβ of the intestine is critical to the phagocytic activity of AMs. Tryptophan and dead *L. plantarum* feeding reversed the lung defense impairment through the IKKβ of the intestine.

### IKKβ of the intestine is critical in antibiotics treatment for maintaining plasma IL-6 levels

To evaluate the role of intestine mucosa IKK activity in antibiotic treatment-induced plasma IL-6 levels, we examined the plasma IL-6 levels in *Vil*-*Cre/Ikkβ*^*F/Δ*^ and *Ikkβ*^*F/Δ*^ mice. The antibiotics treatment significantly increased plasma IL-6 levels in *Ikkβ*^*F/Δ*^ mice compared to the control. The antibiotics only, antibiotic with tryptophan, and antibiotic with dead *L. plantarum* treatments did not change plasma IL-6 levels in *Vil*-*Cre/Ikkβ*^*F/Δ*^ compared the control (Fig. 6b). These results indicate that the IKKβ of the intestine is critical in maintaining IL-6 levels in the plasma during antibiotics treatment.

### The IKKβ of the intestine is critical in the peroxynitrite production of AMs and the inhibitory effects of antibiotic treatment on peroxynitrite production of AMs

To evaluate the involvement of intestinal IKK activity in antibiotic treatment-induced lung defense impairment, we examined the peroxynitrite production of AMs in *Vil*-*Cre/Ikkβ*^*F/Δ*^ and *Ikkβ*^*F/Δ*^ mice. Antibiotics treatment significantly decreased the peroxynitrite production of AMs in *Ikkβ*^*F/Δ*^ mice compared to the control group. However, the antibiotics only, antibiotics with tryptophan, and antibiotics with dead *L. plantarum* feeding treatments did not change the peroxynitrite production of AMs in *Vil*-*Cre/Ikkβ*^*F/Δ*^ mice compared to the control (Fig. 6c). These results indicate that the IKKβ of the intestine is critical in the inhibitory effects of antibiotic treatment on the peroxynitrite production of AMs.

### The IKKβ of the intestine is critical in the effect of antibiotic treatment on Ahr and RELMβ expression

**Fig. 7:**
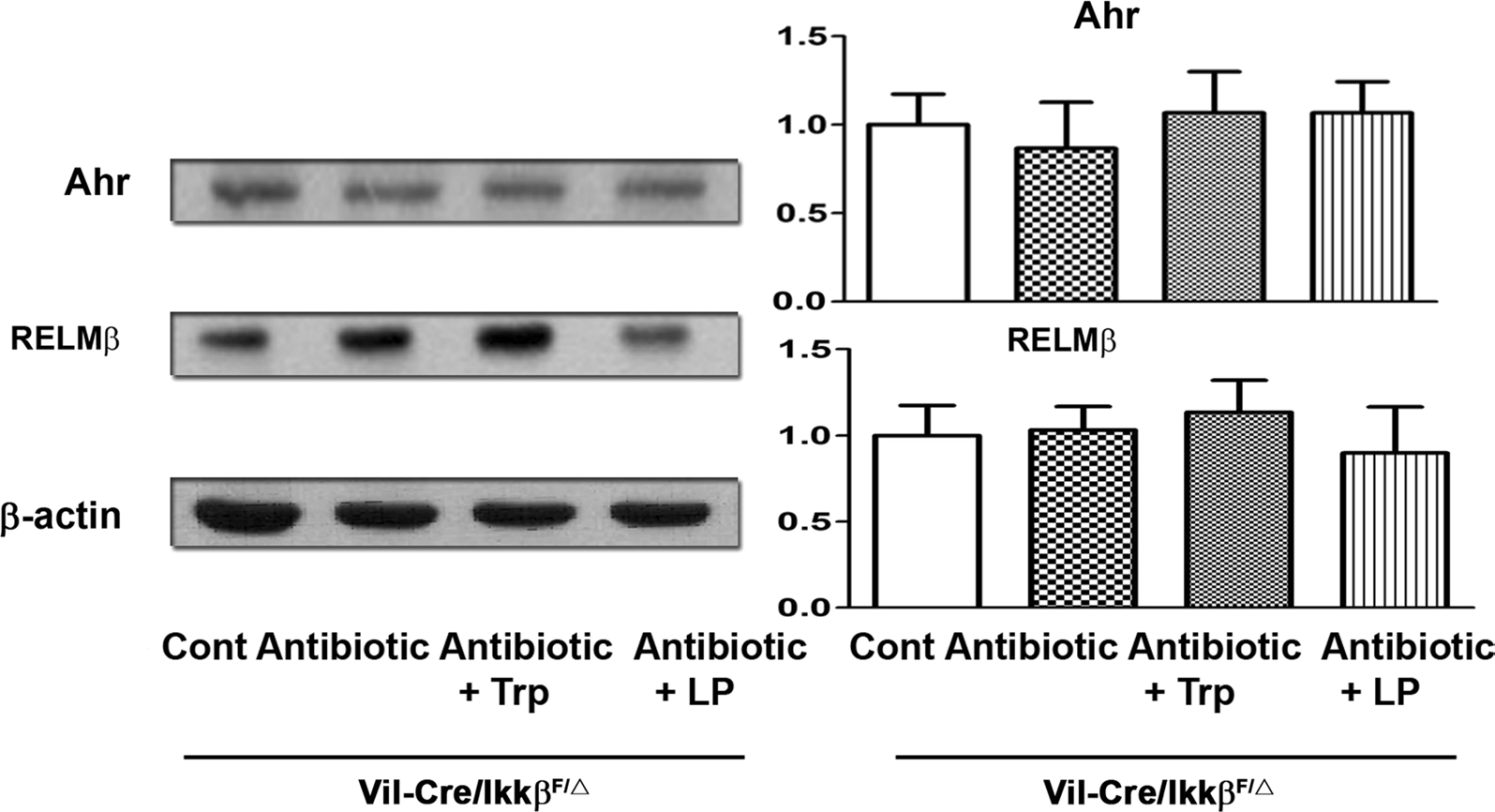
IKKβ of intestine is critical in the Ahr and RELMβ expression of the intestine. Antibiotics, antibiotics with Tryptophan, or antibiotics with dead *L. plantarum* did not change protein expression of Ahr and RELMβ of intestinal mucosa in *Vil*-*Cre/Ikkβ*^*F/Δ*^ mice as compared with the control group. The Ahr, RELMβ, and CRP-ductin were identified by Western immunoblots. Data are presented as mean ± SEM

To assess the involvement of intestinal IKKβ in Ahr and RELMβ expression of the intestine, we examined the protein expression of Ahr and RELMβ of the intestinal mucosa in *Vil*-*Cre/Ikkβ*^*F/Δ*^ and *Ikkβ*^*F/Δ*^ mice. The antibiotics, antibiotics with tryptophan, and antibiotics with dead *L. plantarum* treatments did not change protein expression of Ahr and RELMβ of the intestinal mucosa in *Vil*-*Cre/Ikkβ*^*F/Δ*^ compared to the control (Fig. 7). These results indicate that the IKKβ of the intestine is critical in the effect of antibiotic treatment with or without tryptophan, or dead *L. plantarum* supplementation on Ahr and RELMβ expression of the intestine.

## Discussion

The widespread use of antibiotics increased the emergence of nosocomial infections caused by antibiotic-resistant Gram-negative bacteria in ICU patients, through undefined mechanisms [2, 3]. Here we demonstrated that combined antibiotic treatments led to the impairment of the innate lung immune defenses against infection by *P. aeruginosa*. There are multiple effects that account for this impairment. First, the antibiotic treatment increased the *P. aeruginosa* pneumonia-induced bacterial counts in the lung tissue. Second, the antibiotic treatment induced the reduction of phagocytic activity, physiological activity, and peroxynitrite production of the AMs. Third, the antibiotic treatment increased ICAM and IL-6 expression in the lung. Altogether, our data suggest that antibiotic treatments reduces lung defense against pneumonia caused by PA through the reduction of phagocytic activity, physiological activity, and peroxynitrite production of the AMs. Our results highlight the possibility that broad-spectrum antibiotic treatment increases *P. aeruginosa* pneumonia in critically ill patients by reducing lung immune defense mechanisms.

Recent breakthroughs in the understanding of the importance of the gut microbiome in both the maintenance of health and disease etiology have critical implications for respiratory and critical care medicine [15]. Intestinal microbes not only contribute to the local host defenses against infections, but also modulate systemic immune responses (especially the lung) [27]. Aryl hydrocarbon receptor (Ahr) regulates both innate and adaptive immune responses by detecting a variety of small synthetic and natural chemicals, which act as its ligands. Tryptophan metabolites feeding stimulates intestinal mucosa immunity via the aryl hydrocarbon receptors (Ahr) and IL-22 production [10]. Our results suggest that Ahr ligand supplementation enhanced the lung immune defense mechanism by increasing intestinal ROS and peroxynitrite production of AMs. The Ahr ligands supplementation has three main effects. First, it reverses the antibiotic treatment-induced reduction in Ahr expression of the intestinal mucosa. Second, it increased the peroxynitrite production, physiological activity, and phagocytic activity of the AMs. Third, it increased the ROS production of the intestinal mucosa. Moreover, it also reversed the antibiotic-enhanced ICAM and IL-6 expression and PA pneumonia-induced bacterial counts of the lung. Finally, IKKβ depletion in the gut abolished the stimulatory effects of Ahr ligands and dead *L. plantarum* feeding on Ahr expression of the intestine and peoxynitrite production of AMs. These results suggest that Ahr ligand supplementation reverses the antibiotic treatment-induced lung immune defense impairment through activating IKKβ/NF-κB in the intestine.

ROS levels in the gut have been suggested to be closely related to the intestinal barrier function [28]. Our finding of decreased DCFDA levels in the intestinal mucosa in the antibiotic treatment only group suggests that antibiotic treatment decreased the ROS levels in the gut. Tryptophan, indole, and dead *L. plantarum* feeding in mice receiving antibiotic treatment significantly increased the DCFDA levels in the intestinal mucosa. Superoxide and nitric oxide are essential components in the synthesis of microbicidal compounds (e.g., peroxynitrite), in macrophages [23]. Our finding of decreased peroxynitrite production of AMs in the antibiotic only treatment group suggests that antibiotic treatment reduces phagocytic activity of AMs by decreasing the peroxynitrite production of the AMs. Taken altogether, antibiotic treatment impairs lung immune defense through decreasing ROS production in the gut mucosa and the peroxynitrite production of AMs, and through the subsequent reduction of the phagocytic activity of the AMs. Moreover, oral feeding with tryptophan, indole, and dead *L. plantarum* in mice receiving antibiotic treatment significantly increased the peroxynitrite production and phagocytic activity of the AMs. These results indicate that the Ahr ligands enhance the lung immune defense mechanism by increasing the intestinal ROS production and peroxynitrite production of the AMs. Our study demonstrates clearly a gut–lung axis in the antibiotic model, and establishes a likely mechanism for pulmonary immunomodulation through the intestinal mucosa-mediated signaling pathways. The increasing ROS production of intestine, peroxynitrite production, activity, and phagocytic activity of the AMs brought on by Ahr ligands supplementation further provides support for the regulatory mechanism of the gut on the immune defense system in the lung.

We observed that antibiotic treatment significantly increased the plasma IL-6 levels as compared to that in the control group. Tryptophan, dead *L. plantarum*, and indole feeding in mice receiving antibiotic treatment significantly decreased the IL-6 levels in the plasma. Previously, we have found that combined antibiotic treatment after thermal injury induced a substantial tenfold increase of IL-6 levels in the blood compared with the burn group. Oral supplementation with dead *E. coli* or *S. aureus* in the antibiotic treatment group significantly decreased IL-6 levels in the blood compared with thermal injury with antibiotic treatment group [29]. These results suggest that IL-6 plays an important role in the regulatory mechanisms of gut on lung immunity. Antibiotics treatment increased plasma IL-6 levels in *Ikkβ*^*F/Δ*^ mice. However, antibiotics only, antibiotic with tryptophan, and antibiotic with dead *L. plantarum* treatments did not change plasma IL-6 levels in *Vil*-*Cre/Ikkβ*^*F/Δ*^ mice. These results indicate that the IKKβ of the intestine is critical in inducing plasma IL-6 levels during antibiotics treatment.

The involvements of the intestinal IKK activity/NF-κB activation on the lung defense and its related mechanisms have not been well characterized. We used *Vil*-*Cre/Ikkβ*^*F/Δ*^ mice in antibiotic treatment experiment to examine the involvement of intestinal IKK activity/NF-κB activation in the lung defense mechanism. Previously, we have demonstrated that germ-free mice showed a significant decrease of NF-κB binding activity of intestinal mucosa [13]. Our present data showed that antibiotic treatment significantly decreased binding activity of NF-κB and Ahr expression of the intestinal mucosa. Both results suggested that binding activity of NF-κB of the intestinal mucosa is closely related with microbiota in the intestine. IKKβ depletion in the gut abolished the stimulatory effects of Ahr ligands on Ahr expression of the intestine. These results suggest that IKKβ/NF-κB activation plays an important role in microbiota-induced Ahr expression of the intestinal mucosa. *Vil*-*Cre/Ikkβ*^*F/Δ*^ mice demonstrated a significant increase in PA pneumonia-induced bacterial counts in the lung, and a significant decrease of the phagocytic activity of AMs compared to *Ikkβ*^*F/Δ*^ mice. Moreover, the antibiotics treatment significantly decreased the peroxynitrite production of the AMs in *Ikkβ*^*F/Δ*^ mice but not in *Vil*-*Cre/Ikkβ*^*F/Δ*^ mice. These results indicate that the intestinal IKK activity/NF-κB activation of the intestine are crucial for the maintenance of peroxynitrite production, phagocytic activity of AMs, and lung defense against bacterial infection. Ahr can bind the p65 subunit of nuclear factor kappa light chain enhancer of activated β cells (NF-κB), thereby activating the expression of NF-κB [30]. The antibiotics only, antibiotics with tryptophan, or antibiotics with dead *L. plantarum* treatments did not change the protein expression of Ahr and RELMβ of the intestinal mucosa, or the peroxynitrite production of AMs in *Vil*-*Cre/Ikkβ*^*F/Δ*^. These results suggest that antibiotic treatment reduces Ahr expression of the intestine and subsequently the lung defense mechanism through the IKKβ of the intestine. Ahr ligands increased peroxynitrite production of the AMs and lung defense against *P. aeruginosa* infection through IKKβ/NF-κB activation in the intestine. Altogether, we demonstrated that NF-κB activation in the intestine plays an important role in the lung immune defense mechanisms. Stimulation of IKKβ/NF-κB activation of the intestine with Ahr ligands could be a new therapeutic strategy in enhancing lung defense mechanisms in patients receiving antibiotic treatments.

## Conclusions

**Fig. 8:**
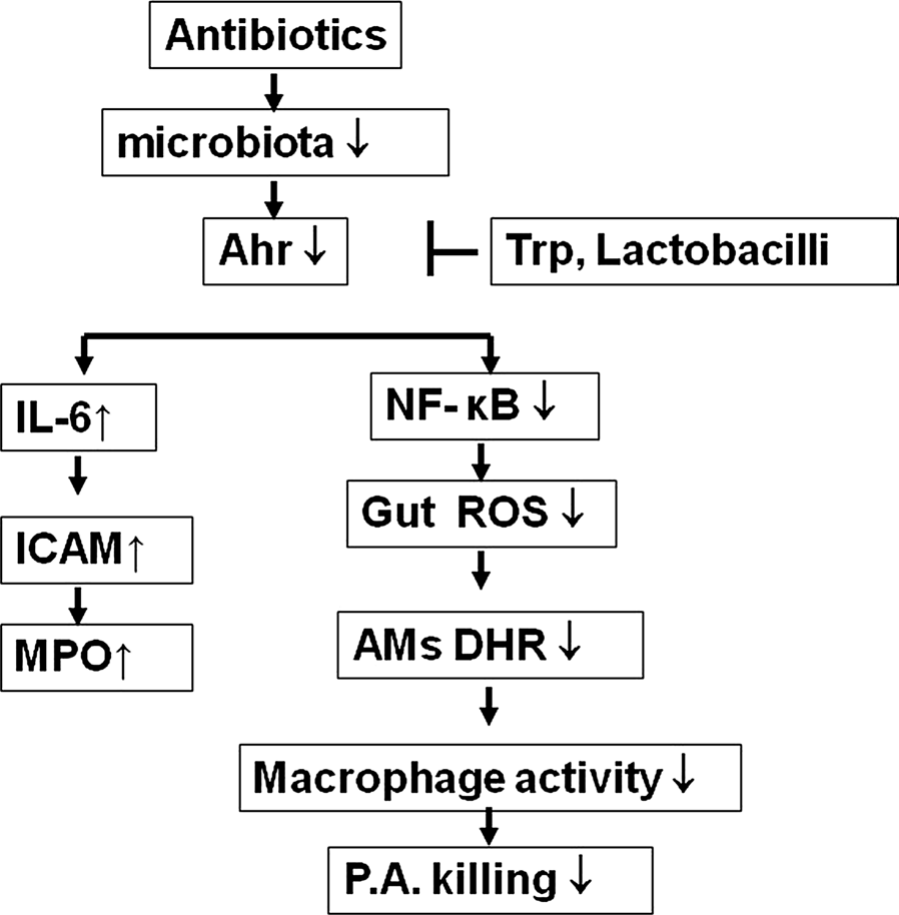
The molecular mechanisms of antibiotic treatment-induced lung defense impairment and roles of Ahr and IKKβ in between

Our study contributes to a better understanding by highlighting the inhibitory effect of antibiotic treatment on lung defense mechanism and the stimulatory effect of Ahr ligands on lung immunity (Fig. 8). Antibiotic treatment decreases the intestinal Ahr expression, ROS production, and NF-κB activation in the intestinal mucosa. The decrease in intestinal NF-κB activation results in the reduction of ROS production and peroxynitrite production of the AMs. This further reduces the phagocytic activity of AMs and lung defense against infection by *P. aeruginosa*. Ahr ligands and dead *L. plantarum* treatments increase Ahr expression, ROS production, and IKKβ/NF-κB activation of the intestinal mucosa, peroxynitrite production, and the phagocytic activity of AMs. Ahr ligands reverse the antibiotic-induced lung defense mechanism through the induction of intestinal IKKβ/NF-κB activation and intestinal ROS production. These observations imply that oral feeding with Ahr ligands or dead *L. plantarum* may be useful to increase the lung immune defense mechanisms in critically ill patients.

## Data Availability

C57BL/6 mice: National Laboratory Breeding and Research Center (NLBRC, Taipei, Taiwan). IKKβ-deficient (*Vil*-*Cre/Ikkβ*^*F/Δ*^) mice and control (*Ikkβ*^*F/Δ*^) mice were generated and transferred from Dr. Karin’s lab. (University of California, San Diego). Ampicillin: Bio Basic Inc. Vancomycin: Hospira. Metronidazole: Sigma. Protein extraction buffer: Sigma-Aldrich. Proteinase inhibitor cocktail: Roche Life Science. Tryptophan: Sigma-Aldrich. Indole: Alfa Aesar. *L. plantaris* (CECT 5713): Dr. Tsai, National Yang-Ming University LPS: Sigma-Aldrich Enhanced chemiluminescence detection reagent: Millipore. Biotinylated anti-mouse, anti-rabbit or anti-goat IgG: GenScript USA Inc. Mouse ELISA kit: eBioscience. Primary antibody for western blotting: R&D Systems. FOS: Sigma-Aldrich. Ketamine hydrochloride: Veterinary Laboratories, Wyeth-Ayerst Canada Inc., Mississauga, ON, Canada). Xylazine: Bayer Inc., Mississauga, ON, Canada.
